# Public libraries and functional disability: A cohort study of Japanese older adults

**DOI:** 10.1016/j.ssmph.2025.101762

**Published:** 2025-02-04

**Authors:** Saeko Otani, Koryu Sato, Naoki Kondo

**Affiliations:** a Department of Social Epidemiology, Graduate School of Medicine and School of Public Health, Kyoto University, Kyoto, Japan; b Faculty of Policy Management, Keio University, Kanagawa, Japan

**Keywords:** Aging, Older adults, Long-term care, Library, Reading habits

## Abstract

This study examined the association between the presence of public libraries and functional disability risk among community-dwelling older adults. We studied 73,138 participants aged 65 years or older in 19 Japanese municipalities using data from the Japan Gerontological Evaluation Study. They were physically and cognitively independent at baseline and followed up between 2013 and 2021 (mean follow-up: 7.3 years). The onset of functional disability was ascertained by linking participants to the public registries of long-term care insurance. The exposures were the number of library books and that of libraries per population in each municipality. During the study period, we observed 16,336 cases (22.3%) of functional disability onset. Our Cox proportional hazards model revealed that the number of library books (hazard ratio [HR] = 0.96, 95% confidence interval [CI]: 0.95–0.97) and that of libraries (HR = 0.52, 95% CI: 0.28–1.00) were associated with the onset of functional disability. The association was consistent even after adjusting for individuals' reading habits and other potential confounders, which suggested the contextual effect of public libraries on older adults’ functional ability. Additionally, the magnitude of association was larger for the younger, women, and people with reading habits than their counterparts. Building new libraries and increasing the number of library books in a community may contribute to lowering the functional disability risk among older adults.

## Highlights

• We studied the association between public libraries and functional disability.
• The number of library books and that of libraries were associated with reduced disability.
• Building new libraries and enhancing their functions may be beneficial for older adults' health.

## 1 Introduction

Functional disability among older adults has become a major public health concern as the global population continues to age. Population aging has started in high-income countries. For example, in Japan, 29.1% of the population is already over 65 years old; at least 6.8 million people (18.9% of the older population) have functional disability; the number of people certified as needing long-term care has increased 2.7 times in the last 20 years (Ministry of HealthLabour and Welfare, 2023). Now, low- and middle-income countries are facing rapid population aging. In the countries of the Association of Southeast Asian Nations, 21.5% of the population has a disability in the activities of daily living (Yau et al., 2022). There is an urgent need to address increasing functional disability worldwide.

Public libraries may be beneficial to realize community healthy aging by nurturing citizens' reading habits and fostering cultural capital (Bourdieu, 1984). Indeed, a previous cohort study has shown that book reading is associated with reduced mortality (Bavishi et al., 2016). Another study has indicated that people who have the habit of reading books, whether or not reading is a hobby, have a lower risk of dementia (Sugita et al., 2021). Existing literature, however, has only focused on individuals’ reading habits. It should be noted that libraries also serve as a place of community engagement, such as hosting cultural events stimulating intellectual curiosity, organizing read-aloud meetings for dementia prevention, and providing volunteer roles to read books to children. A review of the value of public libraries referred to the social aspect of how to improve the quality of life of library users and the community aspect of bridging the social and cultural gaps between users and promoting social participation (Sørensen, 2021). There is a growing body of evidence showing the potential health benefits of community engagement for older adults. Quasi-experimental studies have shown that providing community-dwelling older adults with places for social gathering is associated with improved self-rated health, reduced functional disability, and dementia (Hikichi et al., 2015, Hikichi et al., 2017; Ichida et al., 2013). Additionally, the presence of libraries can promote light exercise and prevent sedentary behavior by going to the library and walking around the building. There are dose-response relationships of reduced functional disability with increasing physical activity and decreasing sedentary behavior among older adults (Du et al., 2024). In short, the presence of public libraries can stimulate cognitive function by encouraging citizens to read books as well as promote social and physical activities in the community. Hence, the contextual effect of public libraries should be explored.

Consequently, this study examined the independent association between public libraries and the risk of functional disability for 7 years, while adjusting for individuals’ reading habits and other socio-demographic confounders using data from a large cohort study of Japanese older adults.

## 2 Methods

### 2.1 Study participants

**Fig. 1:**
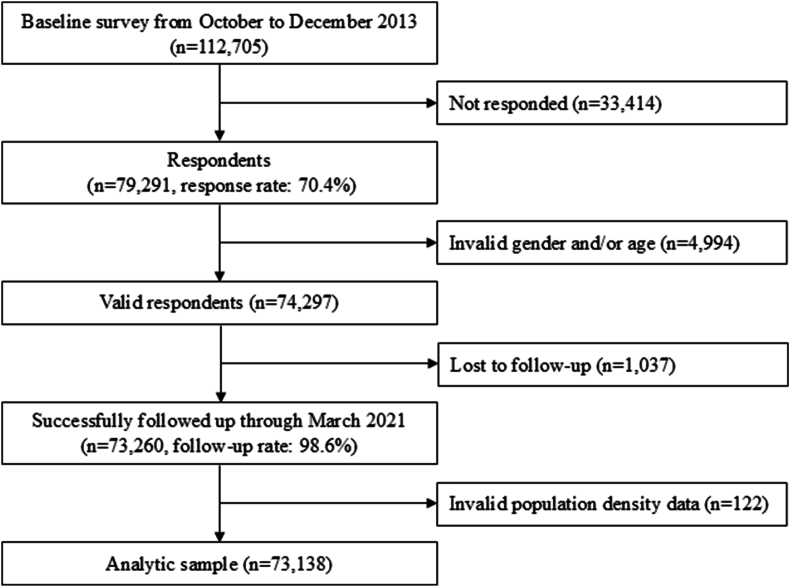
Sample flowchart.

This study used data from the Japan Gerontological Evaluation Study (JAGES), which is an ongoing nationwide cohort study involving those aged 65 years or older who are physically and cognitively independent at baseline (i.e. not certified as needing assistance from public long-term care insurance [LTCI]). Although the JAGES was initiated in 2010, we used the wave of 2013 as the baseline survey considering the balance between the length of the follow-up period and the number of participating municipalities. The survey was conducted in 19 municipalities in Japan from October to December 2013 by distributing self-reported questionnaires. Eligible residents were randomly sampled from 10 large municipalities, and a census of all eligible residents was conducted in the remaining nine smaller municipalities. The questionnaires were mailed to 112,705 residents, of whom 79,291 responded (response rate: 70.4%). Individuals whose sex and age were not confirmed at baseline (n = 4994) were excluded. Of the 74,297 respondents, 73,260 were successfully followed up through March 2021 (mean follow-up: 7.3 years) by linking their data to the public registry of the LTCI (follow-up rate: 98.6%). This study excluded 122 individuals for population density data for their residences were missing; thus, the analytical sample comprised 73,138 participants (Fig. 1).

### 2.2 Outcome

The event of functional disability onsets was ascertained by linking participants to the LTCI registries administrated by each municipality. In Japan, all citizens aged 40 years or older are insured, and official certification is required to receive assistance from the LTCI. In the certification process, a trained municipal investigator visits the applicants’ homes and assesses their physical and cognitive conditions. Based on the home assessment and a documented opinion from a primary physician, the municipal committee composed of experts in health and medical welfare determines whether the applicant needs assistance from the LTCI, and the extent of care requirements are classified into eight levels: Independent (not applicable for assistance), Support 1 and 2, and Care 1 to 5 (higher numbers denote more severe disability). Certification criteria and protocols are standardized nationwide (J. Saito et al., 2022). To examine time to the event using survival analysis, we defined a dichotomized variable of functional disability onsets that takes the value 1 when the participant was newly certified as needing assistance from the LTCI (i.e., Support 1 or higher levels), which is a widely used definition of functional disability (Fujihara et al., 2022; Kanamori et al., 2014; Lu et al., 2023).

### 2.3 Exposure

To capture variations in libraries’ size and function, the present study used the number of library books per population in each municipality as a primary exposure of interest. We assumed that a larger library attracts more people and has a greater impact on community health. Our secondary exposure is the number of libraries, which has less variability than the primary exposure across municipalities. The number of library books and that of libraries in each municipality was provided by the Japan Library Association (2013) and divided by the population of the municipality. Geographical density calculated by the number of libraries divided by the municipal area was also considered as another measure of exposure. We used the municipality as the unit of analysis because many small municipalities have only one public library and each library does not have a specific geographic area of jurisdiction.

### 2.4 Covariates

The following covariates were measured: age (continuous), sex, years of education (≤9 years or ≥10 years), household income (<2.5 million yen, 2.5–7.0 million yen, or ≥7.0 million yen), marital status (married or widowed/divorced/unmarried), and employment status (employed or unemployed). Social participation is assessed using the number of groups that the participant attended at least once a month. Five activities (volunteer, sports, hobby, study or culture, and skills teaching groups) were selected based on a previous study (M. Saito et al., 2017), and thus the score of social participation takes 0–5 points. In addition, we adjusted for the residential population density and the municipal financial strength indexes. The index is an indicator of the fiscal capacity of local governments determined by the Ministry of Internal Affairs and Communications. It is calculated by dividing the revenue by the fiscal demand within the municipality to determine the amount of subsidies from the national government to municipalities. There are many other facilities in communities with high population density and abundant budgets, which may be confounded with the association of interest. We expect that adjustments for these community-level variables can mitigate the potential bias. Model 3 was further adjusted for individual reading habits. Reading habits were assessed using the question, “Do you read books or magazines daily?” The participants were asked to record their responses as yes or no. Reading habits may mediate the relationship between objective and explanatory variables because a large number of books in a library nurtures people's reading habits, which may affect their health. However, this factor was adjusted to examine the contextual effect of libraries in the community, regardless of individuals' reading habits.

### 2.5 Statistical analysis

Cox proportional hazards regression analysis was performed to examine the association between public libraries and the onset of functional disability. We confirmed that the proportional hazards assumption was satisfied using the Schoenfeld residuals test. In Model 1, we showed an unadjusted association. In Model 2, we adjusted for covariates including age, sex, years of education, household income, marital status, employment status, social participation, the municipal financial strength index, and the residential population density. In Model 3, individual reading habits were additionally adjusted for. In addition, we performed the test of interactions and stratified analyses to investigate the heterogeneity of association by age, sex, and reading habits. Assuming that the data were missing at random (i.e., a missing mechanism is related to other variables measured in the same survey for that individual), missing values were predicted using observed covariates including age, sex, years of education, household income, marital status, employment status, social participation, and reading habits. Single imputation was performed using a random forest-based algorithm (Stekhoven & Bühlmann, 2012). All analyses were performed using R version 4.2.1 (R Foundation for Statistical Computing, Vienna, Austria).

## 3 Results

This study involved 73,138 individuals with a mean age of 73.3 years (standard deviation: 6.11), and 53.5% were women (Table 1). Of these, 77.0% reported having a daily reading habit. We observed 16,336 cases (22.3%) of functional disability onset during the study period. The number of library books had a greater variation than that of libraries, given that many small municipalities had only one public library (Table 2). The larger the financial strength index, the stronger the finances of the municipality. Four out of 19 municipalities had an index exceeding 1 and did not receive subsidies from the national government.

**Table 1:** Descriptive statistics for individual-level variables (N = 73,138).

Characteristics	n	%
Age, years		
≤69	23,354	31.9
70-74	21,669	29.6
75-79	15,510	21.2
80-84	8805	12.0
≥85	3800	5.2
Women	39,155	53.5
Years of education, years		
≤9	30,461	41.6
≥10	41,047	56.1
Missing data	1630	2.2
Household income, million yen		
<2.5	25,092	34.3
2.5–7.0	29,846	40.8
≥7.0	6732	9.2
Missing data	11,468	15.7
Marital status		
Married	51,574	70.5
Widowed/Divorced/Unmarried	19,017	26.0
Missing data	2547	3.5
Employment status		
Employed	15,551	21.3
Not working	50,534	69.1
Missing data	7053	9.6
Social participation		
0	33,012	45.1
1	11,486	15.7
2	7732	10.6
3	3504	4.8
4	1279	1.7
5	430	0.6
Missing data	15,695	21.5
Reading habits		
Read	56,305	77.0
Don't read	15,496	21.2
Missing data	1337	1.8
Functional disability	16,336	22.3

**Table 2:** Descriptive statistics for municipality-level variables.

Municipality	Number of libraries	Number of books (thousands)	Population (thousands)	Area (km^2^)	Financial strength index
Higashikawa Town	0	0	8	247.06	0.28
Higasikagura Town	1	78	10	68.64	0.36
Biei Town	1	59	11	677.16	0.19
Towada City	1	128	65	725.67	0.39
Iwanuma City	3	148	44	60.71	0.76
Kashiwa City	18	913	396	114.9	0.92
Niigata City	19	2590	803	726.1	0.72
Chuo City	3	260	30	31.81	0.72
Hayakawa Town	0	0	1	369.86	0.16
Nagoya City	21	4275	2182	326.43	0.98
Hekinan City	3	518	70	35.86	1.03
Tokoname City	1	231	56	55.65	0.96
Tokai City	1	282	109	43.36	1.26
Obu City	1	258	85	33.68	1.04
Chita City	1	312	85	45.76	0.97
Higashiura Town	1	177	49	31.11	0.94
Taketoyo Town	1	222	42	25.82	1.08
Matsuura City	2	135	25	130.38	0.43
Mifune Town	1	17	18	99	0.36

Mean (SD)	4.16 (6.83)	558.05 (1071.86)	215.21 (512.60)	202.58 (240.35)	0.71 (0.34)
Median (IQR)	1 (1–3)	222 (103–297)	49 (21.5–85)	68.64 (39.61–286.75)	0.76 (0.375–0.975)

In the unadjusted Cox model (Model 1 in Table 3), the number of library books was associated with a reduced risk of functional disability (hazard ratio [HR] = 0.95, 95% confidence interval [CI]: 0.93–0.98). This negative association persisted after adjusting for demographic and socioeconomic factors (HR = 0.96, 95% CI: 0.93–0.99 in Model 2). Furthermore, the coefficient of exposure was identical even after adjusting for individuals’ reading habits, suggesting no mediation by reading habits (HR = 0.96, 95% CI: 0.93–0.99 in Model 3). Consistently, the number of libraries divided by the population was associated with decreased onsets of functional disability as shown in Table 4 (HR = 0.52, 95% CI: 0.28–1.00). The geographical density of libraries also indicated a negative association but had a wide confidence interval (HR = 0.74, 95% CI: 0.44–1.25).

**Table 3:** Association between the number of library books and functional disability.

	Model 1	Model 2	Model 3
	HR (95% CI)	HR (95% CI)	HR (95% CI)
Library books/Population	0.95 (0.93–0.98)	0.96 (0.93–0.99)	0.96 (0.93–0.99)
Age		1.14 (1.14–1.15)	1.15 (1.14–1.15)
Sex		1.03 (1.00–1.06)	1.03 (1.00–1.06)
Education		1.02 (0.98–1.06)	1.06 (1.02–1.10)
Household income		0.96 (0.96–0.97)	0.96 (0.96–0.97)
Marital status		0.97 (0.93–1.01)	0.97 (0.93–1.01)
Employment status		0.68 (0.66–0.70)	0.68 (0.66–0.71)
Social Participation		0.85 (0.83–0.87)	0.87 (0.85–0.89)
Financial strength index		1.07 (0.85–1.33)	1.06 (0.84–1.33)
Residential population density		1.00 (1.00–1.00)	1.00 (1.00–1.00)
Reading habits			0.78 (0.75–0.81)

Note: HR = hazard ratio, CI = confidence interval. Municipality-level cluster robust standard errors are estimated.

**Table 4:** Association between library density and functional disability.

Exposure measure	Libraries/Population	Libraries/Area
	HR (95% CI)	HR (95% CI)
Library density	0.52 (0.28–1.00)	0.74 (0.44–1.25)
Age	1.15 (1.14–1.15)	1.15 (1.14–1.15)
Sex	1.03 (0.99–1.06)	1.03 (0.99–1.06)
Education	1.06 (1.02–1.10)	1.06 (1.02–1.10)
Household income	0.97 (0.96–0.97)	0.97 (0.96–0.97)
Marital status	0.97 (0.93–1.00)	0.97 (0.93–1.00)
Employment status	0.68 (0.64–0.71)	0.68 (0.64–0.71)
Social Participation	0.87 (0.85–0.88)	0.87 (0.85–0.88)
Financial strength index	0.95 (0.88–1.03)	0.99 (0.92–1.06)
Residential population density	1.00 (1.00–1.00)	1.00 (1.00–1.00)
Reading habits	0.78 (0.75–0.81)	0.78 (0.75–0.81)

Note: HR = hazard ratio, CI = confidence interval.

Interaction terms and stratified analyses indicated that the associations were heterogeneous. Compared to those aged 75 or older, those under 75 years old exhibited a more beneficial association in Table 5 (HR = 0.95 for age <75 vs. 0.97 for age ≥75). The magnitude of association was larger for women than men as shown in Table 6 (women: 0.95 vs. men: 0.97). Lastly, people with reading habits showed a greater reduction in functional disability than those without reading habits in Table 7 (with reading habits: 0.95 vs. without reading habits: 0.98).

**Table 5:** Stratified analysis by age for the association between the number of library books and functional disability.

Age	All (n = 73,138)	<75 (n = 45,023)	≥75 (n = 28,115)
	HR (95% CI)	HR (95% CI)	HR (95% CI)
Library books/Population	0.93 (0.90–0.96)	0.95 (0.92–0.99)	0.97 (0.95–1.00)
Interaction term (Age ≥75 x library books)	1.05 (1.04–1.06)		
Age	1.14 (1.13–1.14)		
Sex	1.03 (1.00–1.06)	0.88 (0.83–0.94)	1.02 (0.98–1.06)
Education	1.06 (1.03–1.10)	0.90 (0.86–0.95)	1.03 (0.98–1.09)
Household income	0.97 (0.96–0.97)	0.94 (0.92–0.95)	0.99 (0.98–1.00)
Marital status	0.97 (0.93–1.00)	0.86 (0.80–0.92)	0.76 (0.73–0.79)
Employment status	0.69 (0.67–0.71)	0.54 (0.50–0.58)	0.66 (0.63–0.69)
Social Participation	0.86 (0.85–0.88)	0.86 (0.83–0.89)	0.81 (0.79–0.83)
Financial strength index	1.06 (0.84–1.34)	1.03 (0.79–1.35)	0.92 (0.77–1.10)
Residential population density	1.00 (1.00–1.00)	1.00 (1.00–1.00)	1.00 (1.00–1.00)
Reading habits	0.78 (0.75–0.81)	0.75 (0.71–0.79)	0.79 (0.76–0.82)

Note: HR = hazard ratio, CI = confidence interval. Municipality-level cluster robust standard errors are estimated.

**Table 6:** Stratified analysis by sex for the association between the number of library books and functional disability.

Sex	All (n = 73,138)	Men (n = 33,983)	Women (n = 39,155)
	HR (95% CI)	HR (95% CI)	HR (95% CI)
Library books/Population	0.97 (0.94–1.00)	0.97 (0.95–1.00)	0.95 (0.91–0.99)
Interaction term (Female x library books)	0.98 (0.97–1.00)		
Age	0.15 (1.14–1.15)	1.14 (1.14–1.15)	1.15 (1.15–1.16)
Sex	1.09 (1.02–1.17)		
Education	1.06 (1.02–1.10)	1.05 (1.01–1.10)	1.06 (1.02–1.11)
Household income	0.96 (0.96–0.97)	0.95 (0.94–0.96)	0.97 (0.97–0.98)
Marital status	0.97 (0.93–1.01)	0.89 (0.81–0.97)	1.02 (0.98–1.07)
Employment status	0.68 (0.66–0.70)	0.67 (0.64–0.71)	0.70 (0.66–0.75)
Social Participation	0.87 (0.85–0.89)	0.87 (0.85–0.89)	0.87 (0.84–0.89)
Financial strength index	1.06 (0.84–1.33)	1.05 (0.86–1.27)	1.07 (0.82–1.40)
Residential population density	1.00 (1.00–1.00)	1.00 (1.00–1.00)	1.00 (1.00–1.00)
Reading habits	0.78 (0.75–0.81)	0.77 (0.74–0.80)	0.80 (0.75–0.84)

Note: HR = hazard ratio, CI = confidence interval. Municipality-level cluster robust standard errors are estimated.

**Table 7:** Stratified analysis by reading habits for the association between the number of library books and functional disability.

Reading habits	All (n = 73,138)	No (n = 15,515)	Yes (n = 28,115)
	HR (95% CI)	HR (95% CI)	HR (95% CI)
Library books/Population	0.97 (0.94–1.01)	0.98 (0.95–1.01)	0.95 (0.92–0.99)
Interaction term (Reading habits x library books)	0.98 (0.96–1.00)		
Age	1.15 (1.14–1.15)	1.13 (1.13–1.14)	1.15 (1.15–1.16)
Sex	1.03 (1.00–1.06)	1.01 (0.96–1.05)	1.05 (1.01–1.08)
Education	1.06 (1.02–1.10)	1.20 (1.12–1.28)	1.02 (0.98–1.05)
Household income	0.96 (0.96–0.97)	0.98 (0.97–0.99)	0.96 (0.95–0.97)
Marital status	0.97 (0.93–1.01)	0.93 (0.87–1.00)	0.99 (0.95–1.03)
Employment status	0.68 (0.66–0.71)	0.60 (0.54–0.66)	0.72 (0.68–0.76)
Social Participation	0.87 (0.85–0.89)	0.80 (0.75–0.85)	0.88 (0.86–0.90)
Financial strength index	1.06 (0.84–1.33)	1.01 (0.77–1.34)	1.07 (0.86–1.34)
Residential population density	1.00 (1.00–1.00)	1.00 (1.00–1.00)	1.00 (1.00–1.00)
Reading habits	0.84 (0.77–0.91)		

Note: HR = hazard ratio, CI = confidence interval. Municipality-level cluster robust standard errors are estimated.

## 4 Discussion

To our knowledge, this is the first study to examine the longitudinal association between public libraries and functional disability. We found that the number of library books per population was associated with a low risk of functional disability. A hazard ratio of 0.96 could be interpreted as an increase of ten books in the library collection per population corresponding to an approximately 34% reduction (1-0.96^10^) in the onset of functional disability in a municipality. Furthermore, an additional library per population was associated with a 48% reduction in functional disability. Our findings are consistent with previous studies showing the association of book reading with decreased risks of dementia and mortality (Bavishi et al., 2016; Sugita et al., 2021) and add reduced functional disability to the list of potential benefits. The impact of public libraries on functional disability might be larger than that of social participation. A previous study using the JAGES data showed that participation in sports groups and hobby groups once a week was associated with 24% and 17% reductions in the hazard of functional disability for 6 years, respectively (Ide et al., 2023). The exposures were independently associated with reduced functional disability, even after adjusting for individuals’ reading habits. These findings suggest that public libraries may be beneficial for preventing functional disability among community-dwelling older adults.

Interestingly, we found that the magnitude of association was larger for the younger, women, and people with reading habits than their counterparts. Visiting a library requires walking or other forms of light physical activity. Younger older adults are mobile and active in visiting a library more frequently than their counterparts, which could contribute to maintaining physical health and reducing sedentary behavior, thereby lowering the risk of functional disability. Moreover, libraries often function as community centers, offering a variety of programs such as book clubs, workshops, and social gatherings. Women, who participate more frequently in such programs (Ong et al., 2024), could experience greater protective effects due to enhanced social networks and emotional support. It is also plausible that public libraries are more attractive to people with reading habits. For individuals with reading habits, the availability of a library likely enhances the frequency and depth of their cognitive engagement.

Several possible mechanisms link the presence of public libraries with a reduction in functional disability. First, public libraries are community cultural capital that allows citizens to engage in cultural activities. Our findings are consistent with previous studies indicating that cultural engagement (e.g., going to the theatre/museums/concerts) is associated with a lower risk of frailty, disability, and mortality (Fancourt & Steptoe, 2019a, 2019b; Rogers & Fancourt, 2020). Libraries contain a myriad of books in a variety of genres, which stimulates intellectual curiosity. Even if an individual has already decided on a book to borrow, the process of finding that book can sometimes lead to interest in other books. Furthermore, cultural events held at the library help older adults cultivate their skills and interests. Encountering a completely new field in this manner broadens the range of an individual's activities. A greater variety of daily activities is associated with higher cognitive and executive functioning (Lee et al., 2021). Cognitive stimulation that libraries enhance helps older adults maintain cognitive performance and, consequently, can reduce the risk of functional disability.

Second, libraries also provide opportunities for community engagement that is applied to “social prescribing (Drinkwater et al., 2019).” Previous studies have demonstrated that community engagement is associated with a decreased risk of functional disability (Fujihara et al., 2022; Kanamori et al., 2014; Mendes de Leon et al., 2003). Active community engagement can reduce feelings of loneliness and social isolation, which can be protective against the onset of functional disability (Shankar et al., 2017). In some libraries, older people work as volunteers to read to children. A review of intergenerational programs involving interactions between children and older individuals suggested their favorable impacts on older adults’ well-being and self-reported health (Gualano et al., 2018). Having community roles can enhance a sense of *Ikigai* (“what makes life worth living”) that is associated with reduced functional disability (Okuzono et al., 2022). The literature underpins our findings that public libraries, a place for community engagement, can be beneficial to reduce functional disability.

Third, the presence of libraries encourages older adults to be more physically active. The increased frequency of outings to the library enables older adults to engage in physical activity, leading to the maintenance of their physical capacity. Indeed, previous studies have shown that a higher density of neighborhood recreational facilities is associated with increased physical activity, especially among older adults (McCormack et al., 2007; Ranchod et al., 2014). The relationship between the presence of facilities and physical activity is, however, complex. Kaufman et al. (2019) pointed out that facility membership determines the strength of the link between facility density and physical activity. In this regard, public libraries are open for everyone to use and provide opportunities for physical activity without barriers.

The increasing availability of digital books and mobile library services offers new opportunities for older adults to engage in reading and receive intellectual stimulation. These innovations may enhance access to reading materials, particularly for individuals with mobility challenges or those living in remote areas. However, if libraries provide additional benefits beyond intellectual stimulation—such as fostering social interactions or offering physical activities that help maintain older adults’ physical functions—they may prove more advantageous than digital or mobile libraries. Our study did not explore the impacts of digital books or mobile library services, leaving this as a critical area for future research.

Our study has some limitations. First, this study used the number of books as a proxy variable of libraries' size and function; however, as mentioned above, a library does not only serve as a place for reading. If data such as specific services offered by each library, books on loan, and the number of library visits and volunteers were available, we could have studied the library's functions from a more multifaceted perspective. However, no documents summarize information, and such descriptions are sparse, even on individual library websites. Second, facilities that have a similar role to libraries must be considered, such as bookshops and community centers. The presence of bookshops encourages reading, and local community centers are used as places for events and meetings, and some have small reading spaces. To mitigate the potential bias due to community-level confounders, we adjusted for the residential population density and the municipal financial strength indexes, assuming that communities with high population density and abundant budgets have many other facilities. As shown in the results, the association between public libraries and functional disability was very robust even after adjusting for the community-level variable. Third, given the nature of observational studies, we cannot infer a strict causality from these findings. If healthy people prefer to live near libraries, the observed associations can be overstated. Fourth, the generalizability of our findings may be limited to Japanese environments. It should be noted that in Japan, public transportation and walkability are well-developed, which helps older adults to go to the library. In countries like the US, where drive-through libraries are widespread, the results might be different. Finally, explanatory variables were measured at a single point, and we could not consider time-varying variables such as the closure of public libraries and changes in local migration patterns. If the present study could take into account these variables using panel data, we could have better addressed reverse causation.

## 5 Conclusions

Focusing on the environmental factor of libraries rather than the individual behavior of reading, this study found an independent association between public libraries and a reduction in functional disability. Building new libraries and increasing the number of library books in a community may contribute to lowering the functional disability risk among older adults.

## CRediT authorship contribution statement

**Saeko Otani:** Writing – original draft, Methodology, Formal analysis, Data curation, Conceptualization. **Koryu Sato:** Writing – review & editing, Visualization, Validation, Supervision, Funding acquisition. **Naoki Kondo:** Writing – review & editing, Supervision, Resources, Project administration, Funding acquisition.

## Ethical statement

This study was reviewed and approved by the ethics committees of Kyoto University (R3153-2), Chiba University (3442), and Nihon Fukushi University (13–14).

## Funding

This study used data from the Japan Gerontological Evaluation Study, which was supported by 10.13039/501100001691Japan Society for the Promotion of Science (20H00557, 20K10540, 21H03196, 21K17302, 22H00934, 22H03299, 22K04450, 22K13558, 22K17409, 23H00449, 23H03117, 23K21500, 23K27854), 10.13039/501100003478Ministry of Health, Labour and Welfare (19FA1012, 19FA2001, 21FA1012, 22FA2001, 22FA1010, 22FG2001), Japan Science and Technology Agency (JPMJOP1831, JPMJPF2105, JPMJRS24I5), 10.13039/100019434Japan Health Promotion & Fitness Foundation, 10.13039/501100007951Tokyo Medical and Dental University priority research areas grant, and National Research Institute for Earth Science and Disaster Resilience.

## Declaration of interests

The authors have no competing interests to declare.

## Data Availability

Data will be made available on request.
